# Detection of UV-induced cyclobutane pyrimidine dimers by near-infrared spectroscopy and aquaphotomics

**DOI:** 10.1038/srep11808

**Published:** 2015-07-02

**Authors:** Noriko Goto, Gyorgy Bazar, Zoltan Kovacs, Makoto Kunisada, Hiroyuki Morita, Seiichiro Kizaki, Hiroshi Sugiyama, Roumiana Tsenkova, Chikako Nishigori

**Affiliations:** 1 Division of Dermatology, Department of Internal Related, Graduate School of Medicine, Kobe University, Kobe 650-0017, Japan; 2 Biomeasurement Technology Laboratory, Graduate School of Agriculture, Kobe University, Kobe 657-8501, Japan; 3 Institute of Food and Agricultural Product Qualification, Faculty of Agricultural and Environmental Sciences, Kaposvar University, Kaposvar 7401, Hungary; 4 Department of Physics and Control, Corvinus University of Budapest, Budapest 1118, Hungary; 5 Department of Chemistry, Graduate School of Science, Kyoto University, Sakyo, Kyoto 606-8501, Japan

## Abstract

Ultraviolet (UV) radiation causes cellular DNA damage, among which cyclobutane pyrimidine dimers (CPDs) are responsible for a variety of genetic mutations. Although several approaches have been developed for detection of CPDs, conventional methods require time-consuming steps. Aquaphotomics, a new approach based on near-infrared spectroscopy (NIRS) and multivariate analysis that determines interactions between water and other components of the solution, has become an effective method for qualitative and quantitative parameters measurement in the solutions. NIR spectral patterns of UVC-irradiated and nonirradiated DNA solutions were evaluated using aquaphotomics for detection of UV-induced CPDs. Groups of UV-irradiated and nonirradiated DNA samples were classified (87.5% accuracy) by soft independent modeling of class analogy (SIMCA). A precise regression model calculated from NIR water spectral patterns based on UVC doses (r Val = 0.9457) and the concentration of cis-syn cyclobutane thymine dimers (cis-syn T<>Ts; r Val = 0.9993) was developed using partial least squares regression (PLSR), while taking advantage of water spectral patterns, particularly around 1400–1500 nm. Our results suggested that, in contrast to DNA, the formation of cis-syn T<>Ts increased the strongly hydrogen bonded water. Additionally, NIRS could qualitatively and quantitatively detect cis-syn T<>Ts in isolated DNA aqueous solutions upon UVC exposure.

In recent years, the annual incidence of skin cancer has increased dramatically worldwide^1^. Ultraviolet (UV) radiation is undoubtedly one of the most frequent causes of skin cancer^2^. Exposure to UV radiation instantly induces dipyrimidine photoproducts, such as cyclobutane pyrimidine dimers (CPDs), pyrimidine (6-4) pyrimidone photoproducts (6-4PPs), and Dewar valence isomers, in cellular DNA through linkage of two adjacent pyrimidine bases^3^ upon photoexcitation. Many studies have shown that pyrimidine dimers are causes of UV-induced cytotoxicity and mutagenicity. Dipyrimidine photoproducts cause transition-type (pyrimidine-to-pyrimidine) mutations, namely, cytosine (C)-to-thymine (T) or CC-to-TT mutations at dipyrimidine sites^4^,^5^; and these types of mutations are referred to as “UV signature mutations”. CPDs are the major causative molecules inducing cell killing and play an important role in photocarcinogenesis by induction of mutagenicity and immunosuppressive effects^6^,^7^,^8^. In addition, the abundance of CPD production, coupled with slow repair of CPD lesions and inhibition of replication bypass, lead to the development of skin cancer if such lesions occur in crucial genes, such as oncogenes or tumor-suppressor genes^7^,^8^. A high incidence of UV signature mutations has been found in the *TP53* gene in human non-melanoma skin cancers^9^,^10^, and the positions of mutation hotspots in the *TP53* gene are consistent with sites at which CPD removal is particularly slow^11^. A recent study has demonstrated a high frequency of UV signature mutations in the *TRRAP* gene in melanoma cell lines, implying the importance of CPDs in the development of melanoma^12^.

Bulky DNA damage, such as dipyrimidine photoproducts, is repaired by the nucleotide excision repair (NER) pathway^13^, one of the primary DNA repair pathways. Patients with xeroderma pigmentosum (XP), a hereditary disease caused by deficiency in NER, develop non-melanoma skin cancers and cutaneous melanomas of sun-exposed body sites at a 10,000-fold higher frequency and at much younger age than individuals without XP^14^,^15^,^16^. These data indicate that CPDs are closely associated with the development of human skin cancers.

Detection of CPDs can be performed using several methods, including immunofluorescence microscopy^17^, enzyme-linked immunosorbent assays (ELISAs)^18^,^19^, flow cytometry^20^ using monoclonal antibodies^18^, radioimmunoassays (RIAs)^21^, endonuclease-sensitive site (ESS) assays using alkaline agarose gel electrophoresis of DNA incubated with T4 Endonuclease V^22^, and high-performance liquid chromatography with electrospray ionization-tandem mass spectrometry (HPLC-MS/MS)^3^. Detection of thymidine dimers (T<>Ts) using time-resolved infrared spectroscopy in a locked thymine dinucleotide has also been reported^23^. Although most of these conventional methods are useful for detecting and measuring CPDs, they require multistep-procedures for sample preparation or expensive devices and some of them may only be semiquantitative.

Near-infrared spectroscopy (NIRS) applies the near-infrared region (700–2500 nm) of electromagnetic radiation for analyzing various molecules. Recently, qualitative and quantitative models have been developed using NIRS and multivariate analytical methods^24^. NIRS measurements are rapid and nondestructive and do not require expensive devices. Moreover, various NIRS instruments have been developed like contact probes for analysis of human skin^25^. Aquaphotomics focuses on measurement of induced changes in water molecules caused by the solute and described by the respective water spectral patterns as an alternative method for quantitative and qualitative analysis of low concentrations of the solute^26^. In aquaphotomics, water is described as multi-element system that has multidimensional spectra. Water absorbance bands and their spectral patterns can provide important information on water structure and intrinsic interactions between water and other components of solution. Water molecules are always perturbed and take on a variety of structures depending on the environment and the solute, effectively acting as a molecular mirror^27^,^28^. Aquaphotomics uses the spectral patterns of specific structures of water molecules with different strengths and the number of hydrogen bonds detected experimentally or reported previously in the IR range^29^,^30^. Aquaphotomics using NIR spectral patterns has been successfully applied in diverse life science fields such as the diagnosis of mammary gland inflammation by semiquantitative detection of bacteria in cows’ milk^31^,^32^,^33^ or by direct monitoring of mammary glands of cows^34^, identification of human immunodeficiency virus (HIV) infection^35^, diagnosis of prion disease^36^, and forecast of the estrus period in giant pandas^37^.

The objective of this study was to utilize NIRS and aquaphotomics as a new, rapid, simple approach to detect and measure CPDs. NIRS combined with multivariate data analysis was used to detect minimal changes in UV-induced DNA modifications qualitatively and quantitatively. In order to accurately quantify CPDs, we prepared CPDs by irradiating oligonucleotides containing one dithymine site with UVC to produce T<>Ts exclusively.

## Results

### Quantitative evaluation of DNA solutions using NIRS

**Figure 1:**
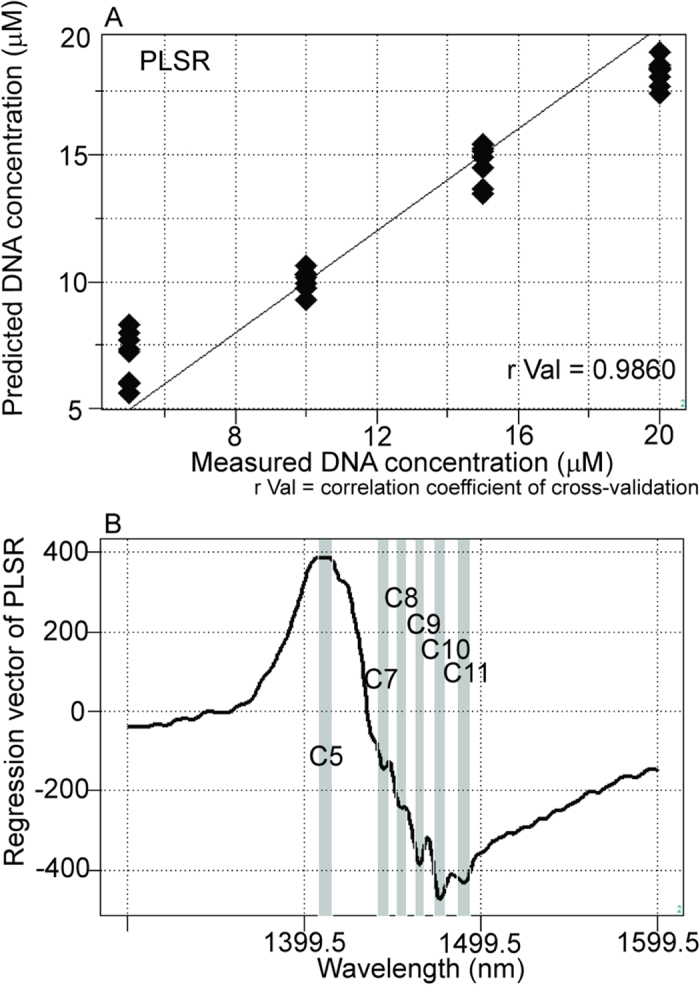
NIRS regression model according to DNA concentration. (**A**) Y-fit for DNA concentration of partial least squares regression (PLSR) with pretreatment by mean centering, smoothing (21 points), OSC (one component), and active class validation. N = 32, number of applied latent variables = 2, r Cal = 0.9978, SEC = 0.3882, r Val = 0.9860, SECV = 1.5131. (**B**) Regression vector of the PLSR calibration model for DNA concentration showing characteristic water peaks at the 1400–1500 nm spectral interval.

First, we assessed whether a regression model for DNA at low concentrations could be developed using NIRS. An accurate NIRS model was fitted to the actual concentration of the DNA solution (5–20 μM) using partial least squares regression (PLSR), as shown in Fig. 1A (r Val = 0.9860, SECV = 0.3882). The result of principal component regression (PCR) also showed sophisticated modeling (Supplementary Fig. S1A; r Val = 0.9985, SECV = 0.3528). Clear peaks were identified at previously described water bands for C5, C7, C8, C9, C10, and C11^26^ (nm) in regression vectors of PLSR and PCR (Fig. 1B and Supplementary Fig. S1B).

### Discriminating Milli-Q water and cis-syn T<>T solutions

**Figure 2:**
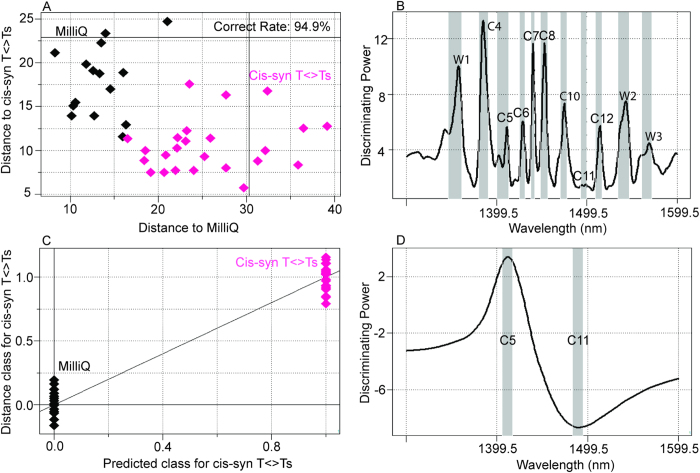
NIRS-based discrimination of Milli-Q water and cis-syn T<>Ts solutions. (**A**) Soft independent modeling of class analogies (SIMCA) using the 1300–1600 nm interval of NIR spectra with mean-centering and smoothing (45 points). Factor # = 2 for samples of Milli-Q water and isolated cis-syn T<>Ts solutions from separate groups (ratio of correctly classified samples = 94.9%). (**B**) Discriminating power of SIMCA showing previously described peaks around 1400–1500 nm. (**C**) Partial least squares-discriminant analysis (PLS-DA) using the 1300–1600 nm interval of NIR spectra with mean-centering, smoothing (45 points), orthogonal signal correction (with one component), and leave-one-out cross-validation; 94.9% of samples were classified correctly in cross-validation. Factor # = 1. (**D**) Regression vector of PLS-DA revealed strong peaks at 1398–1420 nm and 1460–1514 nm.

NIRS was applied to identify pure Milli-Q water and Milli-Q water containing cis-syn T<>Ts (0.77–3.0 μM). Cis-syn T<>Ts were isolated using HPLC with the DNA sequence of 5′-GTAATTAC-3′ irradiated with UVC, which is expected to produce 5′-GTAAT<>TAC-3′. NIR spectra representing Milli-Q water and isolated cis-syn T<>T solutions were very well separated by soft independent modeling of class analogy (SIMCA; Fig. 2A). The model classified 94.9% of the samples correctly. Peaks showing discriminating power (Fig. 2B) were observed at the specific water bands W1, C4, C5, C6, C7, C8, C10, C11, C12, W2, and W3^26^,^38^,^39^ (nm). NIR spectra of Milli-Q water and cis-syn T<>T solutions were also separated by partial least squares-discriminant analysis (PLS-DA; ratio of correctly classified samples: 94.9%; Fig. 2C). The regression vector showed two characteristic water peaks at C5 and C11^26^ (nm) (Fig. 2D).

### Identification of nonirradiated DNA solutions and UVC-irradiated DNA solutions

**Figure 3:**
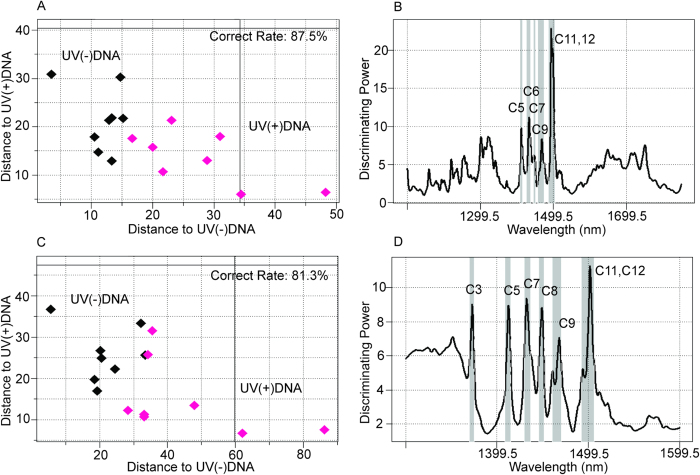
NIRS-based discrimination of irradiated (20 kJ/m^2^ UVC) DNA from nonirradiated DNA in aqueous solutions (20 μM). (**A**) SIMCA using 1100–1850 nm NIR spectra with pretreatment by mean-centering and smoothing (45 points). Factor # = 2. Nonirradiated and UVC-irradiated DNA solutions were distinguished (total ratio of correctly classified samples = 87.5%). (**B**) Discriminating power of SIMCA (1100–1850 nm) showing characteristic peaks in the first overtone region for water (around 1400–1500 nm), with small peaks outside of the 1300–1600 nm interval. (**C**) SIMCA using NIR data representing the first overtone region of water (1300–1600 nm). Pretreatment by mean-centering and smoothing (45 points) were applied. Factor # = 2. Nonirradiated DNA solutions and UVC-irradiated DNA solutions were distinguished (total ratio of correctly classified samples = 81.3%). (**D**) Discriminating power of SIMCA (1300–1600 nm) showed characteristic water peaks around 1400–1500 nm.

Subsequently, NIRS was applied to separate DNA solutions irradiated with UVC 20 kJ/m^2^ from nonirradiated DNA solutions (20 μM) using different wavelength ranges in order to identify regions containing the most relevant information from the UVC irradiation. The SIMCA model classified 87.5% and 81.3% of the irradiated DNA solutions correctly when the spectral intervals of 1100–1850 nm (Fig. 3A) and 1300–1600 nm (Fig. 3C) were used, respectively. The SIMCA interclass distances were 0.9412 and 0.9231 for the two spectral regions, respectively. Spike peaks were mostly observed at C5, C6, C7, C9, C11, and C12^26^ for the spectral range from 1100 to 1850 nm, although small peaks were found outside of the 1300–1600 nm range (Fig. 3B). For the 1300–1600 nm range, strong peaks were observed at C3, C5, C7, C8, C9, C11, and C12^26^ (Fig. 3D). Peaks of regression vectors of PLS-DA were found at C5, C7, C9, C10, and C11^26^ for the 1100–1850 nm range (Supplementary Fig. S2A) and at C5, C7, C9, C10, and C11^26^ for the 1300–1600 nm range (Supplementary Fig. S2B).

### HPLC-based determination of cis-syn T<>Ts in the DNA solutions irradiated with UVC

**Figure 4:**
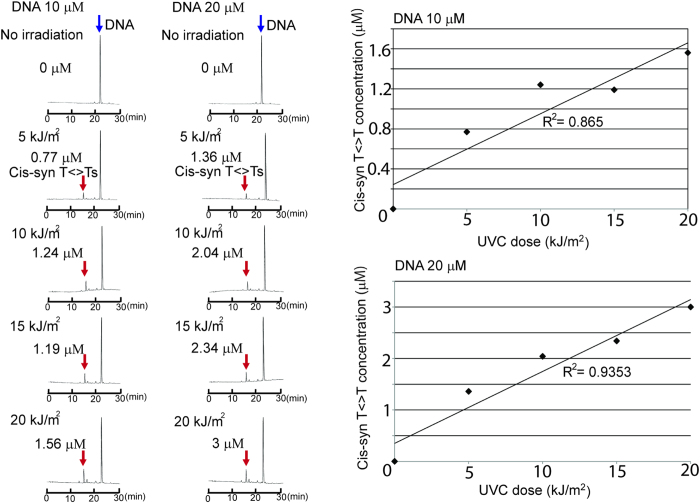
Results of HPLC analysis of the investigated DNA samples. Cis-syn T<>T concentrations in 10 and 20 μM DNA solutions irradiated with UVC (0, 5, 10, 15, or 20 kJ/m^2^) correlated with UVC dose. Linear approximations (R^2^ values) = 0.865 and 0.9353 for 10 and 20 μM DNA solutions, respectively.

In order to confirm the concentration of cis-syn T<>Ts in the investigated DNA solutions irradiated with UVC and to evaluate the correlation with the NIRS model, the cis-syn T<>T concentration of each DNA solution (10 or 20 μM) irradiated with UVC (0, 5, 10, 15, or 20 kJ/m^2^) was determined by means of HPLC-based isolation and quantification. Positive correlations were found between the irradiation dose and the concentration of produced cis-syn T<>Ts (Fig. 4). This result is consistent with previous reports^3^.

### Quantitative analysis of cis-syn T<>Ts using NIRS and aquaphotomics

**Figure 5:**
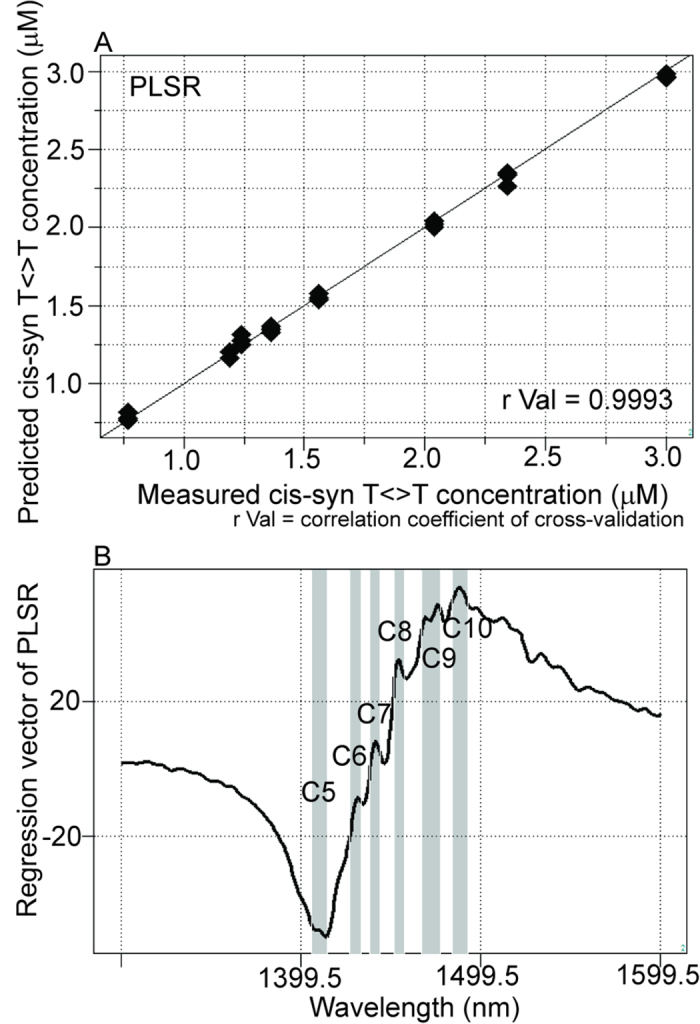
Quantitative analysis of isolated cis-syn T<>Ts using NIRS and HPLC data showing high correlations between cis-syn T<>T concentrations determined by the NIR calibration model and laboratory reference values (0.77–3.0 μM) determined by HPLC. (**A**) Y-fit for cis-syn T<>T concentration of PLSR with pretreatment by mean centering, smoothing (21 points), OSC (one component), and leave-one-out cross-validation. N = 24, number of applied latent variables = 2, r Cal = 0.9993, SEC = 0.0267, r Val = 0.9993, SECV = 0.0308. (**B**) Regression vector for the PLSR calibration model of the cis-syn T<>T concentration revealed characteristic water peaks at 1400–1500 nm.

The quantitative data for cis-syn T<>Ts isolated by HPLC were used as reference data for NIRS calibration. PLSR models were used for quantitative analysis to predict the concentration of isolated CPDs (0.77–3.0 μM) based on the near-infrared spectra of the aqueous samples. Results showed significant correlations and low error (r Val = 0.9993, SECV = 0.0308; Fig. 5A). Definite peaks were observed in the regression vectors at C5, C6, C7, C8, C9, and C10^26^ at approximately 1400–1500 nm. Characteristic water bands indicated relationships between water structures and the dissolved substance (Fig. 5B).

### NIRS regression model dependent on the UVC-irradiation dose

**Figure 6:**
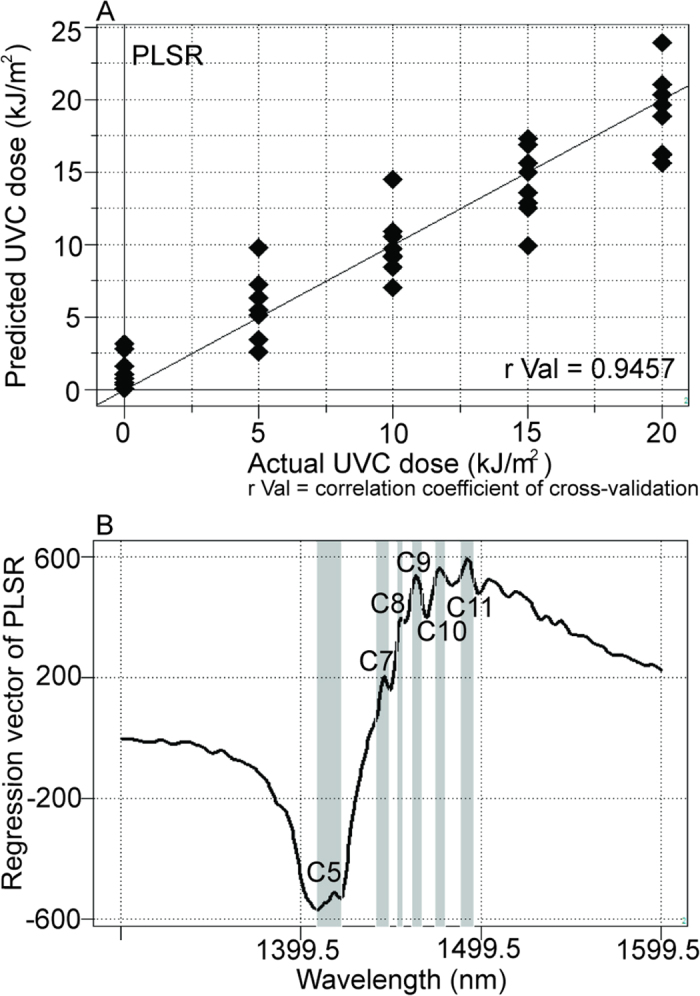
Results of NIRS regression models dependent on irradiated UVC doses showing correlations between the actual doses of UVC irradiation and the levels determined by the NIRS calibration model when DNA samples irradiated with UVC at doses of 0, 5, 10, 15, or 20 kJ/m^2^ were measured in 20 μM aqueous solutions. (**A**) Y-fit of PLSR for UVC doses with pretreatment by mean centering, smoothing (21 points), OSC (one component), and leave-one-out cross-validation (r Val = 0.9457). (**B**) Previously defined water bands assigned in the regression vector of the PLSR calibration model for UVC irradiation doses.

After successful NIRS-based quantitative evaluation of cis-syn T<>Ts, quantitative regression models were developed on NIRS data of irradiated DNA solutions depending on irradiated UVC doses (0, 5, 10, 15, and 20 kJ/m^2^). Results of the accurate NIRS models fitted on actual UVC doses are summarized in Table 1. The results of PLSR for 20 μM DNA solutions irradiated with each dose of UVC are plotted in Fig. 6A (r Val = 0.9457, SECV = 2.3166). Remarkably similar results for PCR (r Val = 0.9550, SECV = 2.1111) were found (Supplementary Fig. S3A). Clear peaks were detected at C5, C7, C8, C9, C10, and C11^26^ in regression vectors for PLSR and PCR (Fig. 6B and Supplementary Fig. S3B).

### NIRS regression model showing the concentrations of the produced cis-syn T<>Ts in irradiated DNA samples

**Figure 7:**
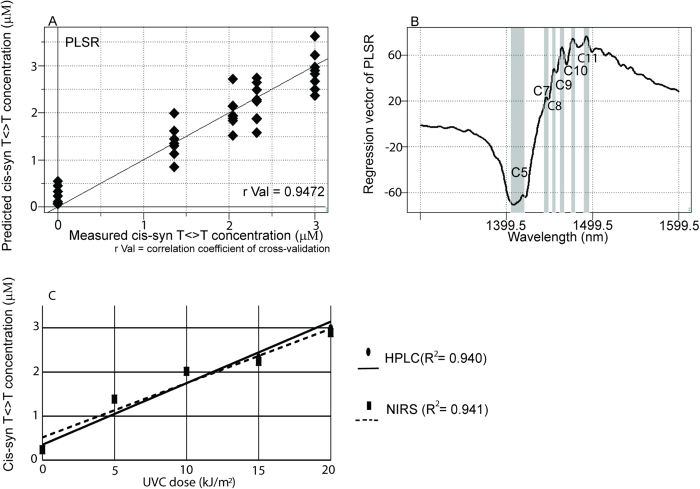
Results of NIRS regression models for the concentrations of cis-syn T<>Ts in DNA solutions showing correlations between the HPLC-based reference data and the NIRS predicted values when DNA samples irradiated with UVC at doses of 0, 5, 10, 15, or 20 kJ/m^2^ were measured in 20 μM aqueous solutions. (**A**) Y-fit of PLSR with mean centering, smoothing (21 points), OSC (one component), and leave-one-out cross-validation for cis-syn T<>T concentrations (r Val = 0.9472). (**B**) Previously defined water bands assigned using the regression vector of the PLSR calibration model for cis-syn T<>T concentrations in DNA solutions. **C**) Graphs plotting the average predicted cis-syn T<>T concentrations using NIRS with PLSR as a function of the UVC dose are shown (R^2^ = 0.941).

Correlations were observed between the irradiated UVC doses and cis-syn T<>T concentrations in DNA samples measured by HPLC (Fig. 4), and successful NIRS calibrations of DNA samples were carried out according to the dose of UVC irradiation (Fig. 6 and Table 1). Next, quantitative NIRS models were fitted to the cis-syn T<>T concentration of the investigated DNA solutions. The cis-syn T<>T concentration determined by HPLC was applied as reference data for the NIRS calibrations. Results of the PLSR and PCR models fitted to the cis-syn T<>T concentration in 20 μM DNA solutions are shown in Fig. 7A (r Val = 0.9472, SECV = 1.7482) and Supplementary Fig. S4A (r Val = 0.9399, SECV = 1.7612). Definite peaks were observed at characteristic water bands (C5, C7, C8, C9, C10, and C11^26^) in the regression vectors for PLSR and PCR (Fig. 7B and Supplementary Fig. S4B). The results of PLSR and PCR achieved in 10 and 20 μM DNA solutions are listed in Supplementary Table S1. Graphs plotting the average predicted cis-syn T<>T concentrations using NIRS with PLSR and PCR as a function of the UVC dose are shown in Fig. 7C (R^2^ = 0.941) and Supplementary Fig. S4C (R^2^ = 0.956), respectively. The results of HPLC determination showed similar correlations between the applied UVC irradiation dose and the generated CPD concentration.

## Discussion

The objective of this study was to evaluate UV-induced DNA damage by NIRS and aquaphotomics based on the detection of pyrimidine dimers, which are key molecules that cause UV-dependent cytotoxicity and mutagenesis and have biological influences on the human body, including induction of photocarcinogenesis. To simplify the experimental system, we produced T<>Ts in DNA by irradiating the oligonucleotide sequence 5′-GTAATTAC-3′ with UVC using germicidal lamps, emitting 240–260 nm; the spectral interval of 1100–1850 nm was used for NIRS analysis. This distant, low-energy NIR wavelength region was chosen to prevent the interference of strong light in the quantification of T<>Ts and for further *in vivo* analysis. We aimed at measuring CPDs directly by HPLC and indirectly as a mirror image of the water molecule system described by the NIR spectral pattern using the concept of aquaphotomics. HPLC has been applied as an established method for quantitative measurement of T<>Ts^3^,^40^.

First, we confirmed that it was possible to evaluate DNA solutions quantitatively (Fig. 1A and Supplementary Fig. 1A). Milli-Q water and cis-syn T<>T solutions were successfully separated based on their near-infrared spectral data (Fig. 2A), and nonirradiated and UVC-irradiated DNA solutions were able to be distinguished using both SIMCA and PLS-DA (Fig. 3A,C). NIRS-based quantitative models for isolated cis-syn T<>T concentrations (Fig. 5A), UVC irradiation doses, and cis-syn T<>T concentrations in DNA samples were developed using PLSR and PCR methods (Fig. 6A, Fig. 7A, Supplementary Fig. S3A, and Supplementary Fig. S4A). PCR develops regression models using principal components derived from spectral data only, while PLSR models generate latent variables that describe the variance of both the spectral and reference values. Both of these chemometric approaches yielded correlations between NIRS predicted data and reference values as measured by HPLC. These two chemometric methods demonstrated the stability of the applied methodology, suggesting that NIRS and aquaphotomics were capable of analyzing CPDs qualitatively and quantitatively.

Regression vectors for PLS-DA, PLSR, and PCR showed two broad peaks (Figs 1B, 2D, 5B, 6B, and 7B; Supplementary Figs. S1B, S2A, S2B, S3B, and S4B) at C5 and C11^26^ (1398–1418 and 1482–1495 nm), covering the areas of S0 (free water) and S4 (water molecules with four hydrogen bonds respectively, Figs 1B, 5B, 6B, and 7B; Supplementary Fig. S1B, S2A, S2B, S3B, and S4B), and several small peaks in between these areas at C5, C7, C8, and C10^26^ (1432–1444, 1448–1454, 1458–1468, and 1402–1482 nm) consistent with S1 (water molecules with one hydrogen bond; water solvation shell, OH-(H_2_O)_4,5_); S2 (water molecules with two hydrogen bonds), and S3 (water molecules with three hydrogen bonds). These peaks formed a spectral pattern that represents the interactions between water molecules and DNA in the studied DNA concentration range. Specifically, water hydrates DNA, thereby losing strongly hydrogen bonded water in the presence of DNA, which results in ion hydration and increasing of weakly H-bonded water and water solvation shell structures^41^. Strong hydrogen bonds are thought to be partially resolved by formation of T<>T dimers after UVC irradiation. One possible explanation for the detectability is the distortion of DNA and the structure of dithymine sites, which may result in slight changes in the interaction with surrounding water molecules, altering the structure of the water matrix. Future studies are needed to determine the validity of this hypothesis. Strong absorption bands at 1692, 1661, and 1631 cm^−1^, indicating double-bond stretching of the two carbonyl groups and the C5 = C6 double-bond of the thymine base, respectively, were found in a previous report^23^. Furthermore, increased absorption was observed at approximately 1465, 1402, and 1320 cm^−1^ for the T<>T lesions^23^. The harmonic band in the NIR region of the aforementioned fundamental bands representing the C5 = C6 double-bond is found between 1488 and 1543 nm^42^; this range appeared many times in our work, with different absorbance ranges observed for DNA samples with and without UVC irradiation. Peaks appeared between 1482 and 1495 nm in the regression vector of the model from the sample of T<>Ts and original DNA without the formation of T<>Ts (Fig. 6), but were not observed in the regression vector of the model from the pure isolated cis-syn T<>T solution (Fig. 5B). However, the weight of the signal was not sufficient for creation of a high-accuracy model. While conventional IR analysis aims to find characteristic bands of the examined component, NIR spectroscopy and aquaphotomics measure changes in water structure in aqueous solutions as molecular vibrational spectral patterns at specific water absorbance bands.

Several types of pretreatments were tested in the present study. Smoothing was applied to reduce the noise of spectral data for all types of methods. When more smoothing points (21 or 45 points) were applied, peaks around 1400–1500 nm remained, and the noise of the entire region decreased. The remaining peaks of discriminating power in SIMCA and regression vectors in PLS-DA, PLSR, and PCR, regardless of the points of smoothing, revealed the significance of the first overtone region of water (1300–1600 nm) and accentuated the validity of the analyses.

All analyses were conducted using NIR spectra both in the range of 1300 to 1600 nm representing the first overtone of water (various vibrational bands of O–H bonds) and in the range of 1100 to 1850 nm representing C–H, N–H, and S–H bonds. In both cases, informative peaks were observed at approximately 1400–1500 nm for the discriminating power of SIMCA and regression vectors of PLS-DA (Fig. 3B, Fig. 3D, Supplementary Fig. S2A, and Supplementary Fig. S2B), demonstrating the importance of spectral information in the range of the first overtone region of water (1300–1600 nm). This phenomenon was also found in our other analyses, suggesting that DNA damage could be detected through changes in the spectral pattern within the characteristic absorbance range of water. The ratio of correctly classified samples in SIMCA was nearly identical for the spectral intervals of 1300–1600 and 1100–1850 nm (Fig. 3A,B).

Quantitative analysis of minor changes in DNA caused by the formation of T<>Ts was difficult since environmental influences, such as humidity and temperature, could cause relatively large spectral fluctuations compared to those arising from the observable molecular changes. OSC was applied to eliminate unwanted spectral effects before PLS-DA, PLSR, and PCR. OSC is regarded as part of the calibration process rather than as a pretreatment and is used to remove unnecessary orthogonal factors and maintain meaningful information^43^,^44^,^45^. Application of OSC significantly improved analyses using PLS-DA, PLSR, and PCR in the present study.

The experiment was carried out under the simplest conditions using DNA (5′-GTAATTAC-3′) containing one dithymine site, which was expected to produce thymine dimers effectively by UVC irradiation. Because the peak absorbance of DNA is around 260 nm, the dimerization of pyrimidines is most effectively produced around 260 nm in a dose-dependent manner^3^,^46^. Conventional knowledge dictates that UV irradiation with longer wavelengths of light predominantly produces oxidatively damaged DNA via indirect mechanisms^47^. However, recent reports have shown that even UVA causes thymine dimers as the primary type of DNA damage in human skin *in vivo*^46^,^48^. Considering that UV light at wavelengths longer than 300 nm reaches the surface of the earth, CPD is presumably produced in human skin during normal daily life at concentrations much higher than previously expected. Some reports have indicated that reactive oxygen species, particularly singlet oxygen, can be measured by NIRS^49^,^50^. However, accounting for the combined effects of thymine dimer production and oxidatively damaged DNA is not easy; thus, future studies are required to develop multivariate NIRS analysis methods for detection of thymine dimers produced by UVA and UVB.

Our ultimate goal is to measure DNA damage caused by UV radiation using NIRS noninvasively in human skin *in vivo*. However, to achieve this goal, several additional factors have to be taken into account. First, in addition to CPDs, 6-4PPs and Dewar valence isomers are produced *in vivo* by solar ultraviolet light. Dewar valence isomers are induced from 6-4PPs mainly by UVA, which accounts for the majority of UV that reaches the surface of the earth^51^. Second, human nuclear DNA has a more complex double helical structure than the single-stranded DNA used in this study. Indeed, human nuclear DNA contains 3 × 10^9^ base pairs, and mitochondrial DNA possesses a circular structure with 15,000–17,000 base pairs^52^. Third, absorption by the stratum corneum and melanin in cells and skin could affect the spectral patterns.

Understanding the influence of these additional factors may not be easy; however, two strategies may be helpful. First, generation of a large spectral database from various sample types may allow the development of a more accurate and advanced regression model by multivariate analysis. Second, subtraction of unnecessary background in NIR spectra may improve the sensitivity of the results, as previously described^53^.

Several *in vivo* application of NIRS have been reported. For example, diagnosis of early-stage mastitis in cows was achieved by analyzing the living body directly using NIRS in real time^34^. Moreover, various NIRS instruments have been developed as contact probes for application to living bodies^25^. In this report, we achieved detection of T<>T dimers qualitatively and quantitatively using NIR spectral data of short single-stranded DNA solutions. However, investigating the influence of all factors described above may make it possible to measure DNA damage caused by UV radiation *in vivo* by applying NIRS with aquaphotomics.

In summary, the qualitative and quantitative detection of DNA damage induced by UVC irradiation was achieved using NIRS with multivariate data analysis based on the aquaphotomics concept. Water was discussed as a molecular mirror that amplifies minute spectral variations caused by the solute. Spectral patterns of water reflected UVC irradiation-induced changes in the DNA structure. This is the first report that investigates photolesion in DNA using NIRS. Potential future applications of NIRS *in vivo* may provide a non-destructive, simple evaluation method of damage caused by UV, contributing substantially to various biomedical fields.

## Methods

### DNA solution samples

A schematic of the study design is shown in Supplementary Fig. S5. The DNA sequence used in this study (5′-GTAATTAC-3′; Life Technologies, Carlsbad, CA) consisted of eight bases and one dipyrimidine site, which presumably produced T<>Ts by UVC irradiation. The stock DNA solution was diluted with Milli-Q water (Direct-Q, Millipore, Molsheim) to a concentration of 100 μM and was then further diluted to each final concentration (15, 30, 45, and 60 μM) in a 500-μL volume in 3.5-cm dishes (Corning, Sigma-Aldrich, St. Louis, MO) as previously described^54^. Each dish was placed on a cooling plate without a cover under a germicidal lamp (GL-15, NEC, Tokyo), which emitted UVC light (240–260 nm, peaking at 253.7 nm). A UVC lamp was used to efficiently produce T<>Ts. The doses of UVC were 0, 5, 10, 15, and 20 kJ/m^2^, as described previously^3^. The dose rate was measured using a UVR-2 instrument (Topcon, Tokyo). After UVC exposure, 1 mL Milli-Q water was added to the experimental DNA solutions in order to reach the volume required for NIRS measurements. The final DNA concentrations were 5, 10, 15, and 20 μM (0.0120, 0.0241, 0.0361, and 0.0482 g/mL). Nine batches of samples were prepared on different days according to the above-described sample preparation protocol.

### HPLC analysis

Since the formation of cis-syn T<>Ts is predominant under our experimental conditions^3^, only cis-syn T<>Ts were measured by HPLC-MS/MS. For HPLC analysis, representative samples (DNA concentrations: 10 and 20 μM; UVC doses: 0, 5, 10, 15, and 20 kJ/m^2^) were prepared in duplicate, and one sample was subjected to NIRS analysis. HPLC analysis was then carried out using a Jasco PU-980 HPLC system and a Chemcobond 5-ODS-H column (4.6 × 150 mm; Chemco Scientific, Osaka) with 50 mM ammonium formate solvent containing 3–10% acetonitrile in a linear gradient at a flow rate of 1.0 mL/min for 30 min at 40 °C. Cis-syn T<>T dimers (5′-GTAAT<>TAC-3′) were isolated using HPLC and identified using electrospray ionization time-of-flight mass spectrometry (ESI-TOF MS) with a Bruker Bio TOF II system (Bruker Daltonics, Billerica, MA).

### NIRS detection

NIRS measurements were performed on eight batches. Each batch was measured within 12 h after sample preparation using a FOSS-XDS spectrometer (FOSS NIRSystems, Inc., Hoganas, Sweden) equipped with a Rapid Liquid Analyzer module. The samples (1 mL of each) were added into 1-mm open-top liquid cuvettes and placed into a temperature-controlled cuvette holder (30 °C), where each sample was incubated for 90 s for tempering before scanning. Acquisition of absorbance values (logT^−1^) was performed with VISION 3.5 software (FOSS NIRSystems, Inc.). The transmittance spectrum of each sample was recorded in the entire spectral range (400–2500 nm), with 0.5-nm steps. A reference spectrum was recorded before analysis of each sample. Milli-Q water was scanned first, followed by the DNA solutions in random order. The spectrum of Milli-Q water was recorded again after every five samples. When a sample was removed from the cuvette, the cuvette was washed first with Milli-Q water and then with 500 μL of the next sample.

### Cis-syn T<>T solution samples and detection by NIRS

DNA containing cis-syn T<>Ts (5′-GTAAT<>TAC-3′) were isolated from the representative DNA solution samples (DNA concentration: 10 or 20 μM; UVC: 0, 5, 10, 15, or 20 kJ/m^2^) using HPLC and were freeze dried. Next, 1.5 mL Milli-Q water was added to each freeze-dried cis-syn T<>T sample just prior to NIRS measurement. Each cis-syn T<>T sample was presented for NIRS scanning twice, as previously described.

### Data analysis

Spectra were imported into the Pirouette 4.0 spectral analytical program (Infometrics, Inc., Woodinville, WA), which was used for data transformation and processing for the spectra intervals of 1100–1850 and 1300–1600 nm. Principal component analysis (PCA) was used to detect spectral outliers. PCA-based SIMCA and PLS-DA were applied to build supervised multiple classification models for qualitative analyses. SIMCA models were evaluated based on the number of misclassifications, level of interclass distances, and discriminating power. PLS-DA models were assessed based on the number of misclassifications and the regression vectors. Quantitative models were developed using PLSR and PCR^24^. The precision and accuracy of the PLSR and PCR methods were evaluated by determining the correlation coefficient (r) of calibration, cross-validation, standard error of calibration (SEC), standard error of cross-validation (SECV), and optimum number of latent variables needed for lowest SEC and SECV^24^. Mean centering and smoothing with varying numbers of points were applied as spectral treatments. OSC was used with one component in order to decrease spectral effects that were independent of the investigated parameters. OSC was applied in PLS-DA, PLSR, and PCR. Leave-one-out cross-validation and active class validation were used for testing the accuracy of the developed PLS-DA, PLSR, and PCR models. In order to avoid overfitting, the maximum number of factors was determined in one-tenth of the total number of samples in the model. The discriminating powers of SIMCA and regression vectors of PLS-DA, PLSR, and PCR models were evaluated to describe the impact of the spectral regions. The ranges of peaks were described as follows: C1 (1336–1348 nm: ν3, H_2_O asymmetric stretching vibration), C2 (1360–1366 nm: water solvation shell, OH-(H_2_O)_1,2,4_), C3 (1370–1376 nm: ν1 + ν3, symmetrical stretching fundamental vibration and H_2_O asymmetric stretching vibration), C4 (1380–1388 nm: water solvation shell, OH-(H_2_O)_1,4_ and superoxide, O_2_-(H_2_O)_4_), C5 (1398–1418 nm: S0, free water and free OH-), C6 (1421–1430 nm: H-OH bend and O…O), C7 (1432–1444 nm: S1), C8 (1448–1454 nm: water solvation shell, OH-(H_2_O)_4,5_), C9 (1458–1468 nm: S2), C10 (1472–1482 nm: S3), C11 (1482–1495 nm: S4), C12 (1506–1516 nm: ν1, ν2, symmetrical stretching fundamental vibration, and doubly degenerate bending fundamental), W1 (1342–1360 nm: H_15_O_7_, H_13_O_6_, H_11_O_5_ + free OH stretch), W2 (1536–1546 nm: H_2_O intermolecular bend angling), and W3 (1565–1586 nm: H_15_O_7_ + H-bonded OH stretch). C1–C12 were defined based on previous literature^26^. W1–W3^38^,^39^ were named expediently. “S” describes number of hydrogen-bonded water molecules.

## Additional Information

**How to cite this article**: Goto, N. *et al.* Detection of UV-induced cyclobutane pyrimidine dimers by near-infrared spectroscopy and aquaphotomics. *Sci. Rep.* **5**, 11808; doi: 10.1038/srep11808 (2015).

## Supplementary Material

Supplementary Information

## Figures and Tables

**Table 1:** Calibration and cross-validation results of PLSR and PCR models for the UVC dose at each DNA concentration.

	DNA concentration							
N	5 μM		10 μM		15 μM		20 μM	
	39		39		39		39	
Model	PLSR	PCR	PLSR	PCR	PLSR	PCR	PLSR	PCR
**Factor #**	3	3	2	2	2	2	2	3
**r Cal**	0.9585	0.9583	0.8821	0.8806	0.9693	0.9692	0.9600	0.9635
**SEC**	2.1418	2.1469	3.4878	3.5098	1.8341	1.8343	2.0727	2.0097
**r Val**	0.9427	0.9453	0.8558	0.8576	0.9649	0.9654	0.9457	0.9550
**SECV**	2.3744	2.3226	3.6835	3.6605	1.8805	1.8680	2.3166	2.1111

N = sample number, Factor # = number of latent variables, r Cal = correlation coefficient of calibration, SEC = standard error of calibration, r Val = correlation coefficient of cross-validation, SECV = standard error of cross-validation.

## References

[b1] WHO. 2014, *Solar ultraviolet radiation: Global burden of disease from ultraviolet radiation 2006.* Available at: http://www.who.int/uv/publications/solaradgbd/ (Accessed: August 18, 2014).

[b2] Budden T. & Bowden N. A. The role of altered nucleotide excision repair and UVB-induced DNA damage in melanomagenesis. Int. J. Mol. Sci. 14, 1132–1151 (2013). 23303275 10.3390/ijms14011132 PMC3565312

[b3] Douki T., Court M., Sauvaigo S., Odin F. & Cadet J. Formation of the main UV-induced thymine dimeric lesions within isolated and cellular DNA as measured by high performance liquid chromatography-tandem mass spectrometry. J. Biol. Chem. 275, 11678–11685 (2000). 10766787 10.1074/jbc.275.16.11678

[b4] Todd P. A. & Glickman B. W. Mutational specificity of UV light in Escherichia coli: indications for a role of DNA secondary structure. Proc. Natl. Acad. Sci. USA 79, 4123–4127 (1982). 7051003 10.1073/pnas.79.13.4123 PMC346589

[b5] You Y. H., Szabo P. E. & Pfeifer G. P. Cyclobutane pyrimidine dimers form preferentially at the major p53 mutational hotspot in UVB-induced mouse skin tumors. Carcinogenesis 21, 2113–2117 (2000). 11062176 10.1093/carcin/21.11.2113

[b6] Nishigori C. *et al.* Evidence that DNA damage triggers interleukin 10 cytokine production in UV-irradiated murine keratinocytes. Proc. Natl. Acad. Sci. USA 93, 10354–10359 (1996). 8816804 10.1073/pnas.93.19.10354 PMC38388

[b7] Nishigori C. Cellular aspects of photocarcinogenesis. Photochem. Photobiol. Sci. 5, 208–214 (2006). 16465307 10.1039/b507471a

[b8] Nishigori C., Hattori Y. & Toyokuni S. Role of reactive oxygen species in skin carcinogenesis. Antioxid. Redox Signal. 6, 561–570 (2004). 15130282 10.1089/152308604773934314

[b9] Brash D. E. *et al.* A role for sunlight in skin cancer: UV-induced p53 mutations in squamous cell carcinoma. Proc. Natl. Acad. Sci. USA 88, 10124–10128 (1991). 1946433 10.1073/pnas.88.22.10124 PMC52880

[b10] Moles J. P. *et al.* p53 gene mutations in human epithelial skin cancers. Oncogene 8, 583–588 (1993). 8437842

[b11] Tornaletti S. & Pfeifer G. P. Slow repair of pyrimidine dimers at p53 mutation hotspots in skin cancer. Science 263, 1436–1438 (1994). 10.1126/science.8128225 8128225

[b12] Wei X. *et al.* Exome sequencing identifies GRIN2A as frequently mutated in melanoma. Nat. Genet. 43, 442–446 (2011). 21499247 10.1038/ng.810 PMC3161250

[b13] Setlow R. B. & Carrier W. L. The disappearance of thymine dimers from DNA: an error-correcting mechanism. Proc. Natl. Acad. Sci. USA 51, 226–231 (1964). 14124320 10.1073/pnas.51.2.226 PMC300053

[b14] Masaki T. *et al.* High frequency of PTEN mutations in nevi and melanomas from xeroderma pigmentosum patients. Pigment Cell Melanoma Res. 27, 454–464 (2014). 24483290 10.1111/pcmr.12226 PMC6309892

[b15] Cleaver J. E. Defective repair replication of DNA in xeroderma pigmentosum. Nature 218, 652–656 (1968). 5655953 10.1038/218652a0

[b16] Bradford P. T. *et al.* Cancer and neurologic degeneration in xeroderma pigmentosum: long term follow-up characterises the role of DNA repair. J. Med. Genet. 48, 168–176 (2011). 21097776 10.1136/jmg.2010.083022 PMC3235003

[b17] Nakagawa A. *et al.* Three-dimensional visualization of ultraviolet-induced DNA damage and its repair in human cell nuclei. J. Invest. Dermatol. 110, 143–148 (1998). 9457909 10.1046/j.1523-1747.1998.00100.x

[b18] Mori T. *et al.* Simultaneous establishment of monoclonal antibodies specific for either cyclobutane pyrimidine dimer or (6-4)photoproduct from the same mouse immunized with ultraviolet-irradiated DNA. Photochem. Photobiol. 54, 225–232 (1991). 1780359 10.1111/j.1751-1097.1991.tb02010.x

[b19] Santella R. M. Immunological methods for detection of carcinogen-DNA damage in humans. Cancer Epidemiol. Biomarkers Prev. 8, 733–739 (1999). 10498391

[b20] Rouget R., Auclair Y., Loignon M., Affar el B. & Drobetsky E. A. A sensitive flow cytometry-based nucleotide excision repair assay unexpectedly reveals that mitogen-activated protein kinase signaling does not regulate the removal of UV-induced DNA damage in human cells. J. Biol. Chem. 283, 5533–5541 (2008). 18093981 10.1074/jbc.M706257200

[b21] Mitchell D. L. Quantification of photoproducts in mammalian cell DNA using radioimmunoassay. Methods Mol. Biol 314, 239–249 (2006). 16673886 10.1385/1-59259-973-7:239

[b22] Higgins K. M. & Lloyd R. S. Purification of the T4 endonuclease V. Mutat. Res. 183, 117–121 (1987). 3547104 10.1016/0167-8817(87)90053-8

[b23] Schreier W. J., Kubon J., Clivio P., Zinth W. & Gilch P. DNA photodamage: Study of cyclobutane pyrimidine dimer formation in a locked thymine dinucleotide. Spectroscopy 24, 309–316 (2010).

[b24] Næs T., Isaksson T., Fearn T. & Davies T. A user-friendly guide to multivariate calibration and classification, Vol. 6, (NIR Publications: Chichester, 2002).

[b25] Arimoto H., Egawa M. & Yamada Y. Depth profile of diffuse reflectance near-infrared spectroscopy for measurement of water content in skin. Skin Res. Technol. 11, 27–35 (2005). 15691256 10.1111/j.1600-0846.2005.00093.x

[b26] Tsenkova R. Introduction: Aquaphotomics: dynamic spectroscopy of aqueous and biological systems describes peculiarities of water. J. Near Infrared Spec. 17, 303–313 (2009).

[b27] Collins J. R. Change in the infra-red absorption spectrum of water with temperature. Phys. Rev. 26, 771–779 (1925).

[b28] Segtnan V. H., Šašić Š., Isaksson T. & Ozaki Y. Studies on the structure of water using two-dimensional near-infrared correlation spectroscopy and principal component analysis. Anal. Chem. 73, 3153–3161 (2001). 11467567 10.1021/ac010102n

[b29] Weber J. M., Kelley J. A., Nielsen S. B., Ayotte P. & Johnson M. A. Isolating the spectroscopic signature of a hydration shell with the use of clusters: superoxide tetrahydrate. Science 287, 2461–2463 (2000). 10741960 10.1126/science.287.5462.2461

[b30] Robertson W. H., Diken E. G., Price E. A., Shin J. W. & Johnson M. A. Spectroscopic determination of the OH- solvation shell in the OH-.(H2O)n clusters. Science 299, 1367–1372 (2003). 12543981 10.1126/science.1080695

[b31] Tsenkova R. *et al.* Near-infrared spectroscopy for dairy management: measurement of unhomogenized milk composition. J. Dairy Sci. 82, 2344–2351 (1999). 10575600 10.3168/jds.S0022-0302(99)75484-6

[b32] Tsenkova R. *et al.* Near infrared spectra of cows' milk for milk quality evaluation: disease diagnosis and pathogen identification. J. Near Infrared Spec. 14, 363–370 (2006).

[b33] Morita H., Kuroki S., Ikuta K. & Tsenkova R. Real-time near infrared spectral monitoring of mammary gland for inflammation diagnosis in dairy cows. J. Near Infrared Spec. 21, 427–434 (2013).

[b34] Shinzawa H., Morita S., Ozaki Y. & Tsenkova R. New method for spectral data classification: two way moving window principal component analysis. Appl. Spectr. 60, 884–891 (2006). 10.1366/000370206778062020 16925924

[b35] Sakudo A. *et al.* A novel diagnostic method for human immunodeficiency virus type-1 in plasma by near-infrared spectroscopy. Microbiol. Immunol. 49, 695–701 (2005). 16034213 10.1111/j.1348-0421.2005.tb03648.x

[b36] Tsenkova R., Iordanova I. K., Toyoda K. & Brown D. R. Prion protein fate governed by metal binding. Biochem. Biophys. Res. Commun. 325, 1005–1012 (2004). 15541389 10.1016/j.bbrc.2004.10.135

[b37] Kinoshita K. *et al.* Spectral pattern of urinary water as a biomarker of estrus in the giant panda. Sci. Rep. 2, (2012). 10.1038/srep00856 PMC3504474 23181188

[b38] Headrick J. M. *et al.* Spectral signatures of hydrated proton vibrations in water clusters. Science 308, 1765–1769 (2005). 15961665 10.1126/science.1113094

[b39] Mizuse K. & Fujii A. Characterization of a solvent-separated ion-radical pair in cationized water networks: infrared photodissociation and ar-attachment experiments for water cluster radical cations (H2O)n+ (n = 3–8). J. Phys. Chem. A 117, 929–938 (2013). 23330841 10.1021/jp311909h

[b40] Douki T. & Cadet J. Individual determination of the yield of the main UV-induced dimeric pyrimidine photoproducts in DNA suggests a high mutagenicity of CC photolesions. Biochemistry 40, 2495–2501 (2001). 11327871 10.1021/bi0022543

[b41] Kojic D. *et al.* Water confined in the local field of ions. Chem. Phys. Chem. 15, 4077–4086 (2014). 25284338 10.1002/cphc.201402381

[b42] Workman J. Handbook of organic compounds : NIR, IR, ramon, and UV-Vis spectra featuring polymers and surfactants. Vol. 2, UV-Vis and NIR spectra. (Academic Press: San Diego, London, 2001).

[b43] Trygg J. & Wold S. Orthogonal projections to latent structures (O-PLS). J. Chemometrics 16, 119–128 (2002).

[b44] Wold S., Antti H., Lindgren F. & Öhman J. Orthogonal signal correction of near-infrared spectra. Chemometr. Intell. Lab. 44, 175–185 (1998).

[b45] Fearn T. OSC and OPLS. NIR News 23, 19–20 (2012).

[b46] Douki T., Reynaud-Angelin A., Cadet J. & Sage E. Bipyrimidine photoproducts rather than oxidative lesions are the main type of DNA damage involved in the genotoxic effect of solar UVA radiation. Biochemistry 42, 9221–9226 (2003). 12885257 10.1021/bi034593c

[b47] Kuluncsics Z., Perdiz D., Brulay E., Muel B. & Sage E. Wavelength dependence of ultraviolet-induced DNA damage distribution: involvement of direct or indirect mechanisms and possible artefacts. J. Photochem. Photobiol. B 49, 71–80 (1999). 10365447 10.1016/S1011-1344(99)00034-2

[b48] Mouret S. *et al.* Cyclobutane pyrimidine dimers are predominant DNA lesions in whole human skin exposed to UVA radiation. Proc. Natl. Acad. Sci. USA 103, 13765–13770 (2006). 16954188 10.1073/pnas.0604213103 PMC1564232

[b49] Umezawa N. *et al.* Participation of reactive oxygen species in phototoxicity induced by quinolone antibacterial agents. Arch. Biochem. Biophys. 342, 275–281 (1997). 9186488 10.1006/abbi.1997.0124

[b50] Arakane K. *et al.* Singlet oxygen (1 delta g) generation from coproporphyrin in Propionibacterium acnes on irradiation. Biochem. Biophys. Res. Commun. 223, 578–582 (1996). 8687438 10.1006/bbrc.1996.0937

[b51] Perdiz D. *et al.* Distribution and repair of bipyrimidine photoproducts in solar UV-irradiated mammalian cells. Possible role of Dewar photoproducts in solar mutagenesis. J. Biol. Chem. 275, 26732–26742 (2000). 10827179 10.1074/jbc.M001450200

[b52] Copeland W. C. & Longley M. J. Mitochondrial genome maintenance in health and disease. DNA repair (Amst) 19, 190–198 (2014). 24780559 10.1016/j.dnarep.2014.03.010 PMC4075028

[b53] Tsenkova R., Meilina H., Kuroki S. & Burus D. Near infrared spectroscopy using short wave lengths and leave-one-cow-out cross-validation for quantification of somatic cells in milk. J. Near Infrared Spec. 17, 345–351 (2009).

[b54] Kunisada M. *et al.* Hydrochlorothiazide enhances UVA-induced DNA damage. Photochem. Photobiol. 89, 649–654 (2013). 23331297 10.1111/php.12048

