# Supplementary material for: Detection of UV-induced cyclobutane pyrimidine dimers by near-infrared spectroscopy and aquaphotomics
Source: Sci Rep. 2015 Jul 2;5:11808. doi: 10.1038/srep11808 (PMC4488872; doi:10.1038/srep11808)
Supplement: Supplementary Information [file srep11808-s1.pdf]

Supplementary information

Detection of UV-induced cyclobutane pyrimidine dimers by near-infrared spectroscopy and aquaphotomics

Noriko Goto[1], Gyorgy Bazar[2, 3], Zoltan Kovacs[2, 4], Makoto Kunisada[1], Hiroyuki Morita[2], Seiichiro Kizaki[5], Hiroshi Sugiyama[5], Roumiana Tsenkova[2*]), and Chikako Nishigori[1*])

[1]Division of Dermatology, Department of Internal Related, Graduate School of Medicine, Kobe University, Kobe 650-0017, Japan

[2]Biomeasurement Technology Laboratory, Graduate School of Agriculture, Kobe University, Kobe 657-8501, Japan

[3]Institute of Food and Agricultural Product Qualification, Faculty of Agricultural and Environmental Sciences, Kaposvar University, Kaposvar 7401, Hungary

[4]Department of Physics and Control, Corvinus University of Budapest, Budapest 1118, Hungary

[5]Department of Chemistry, Graduate School of Science, Kyoto University, Sakyo, Kyoto

606-8501, Japan

Seiichiro Kizaki, Hiroshi Sugiyama

*) Correspondence and requests for materials should be addressed to

RT (rtsen@kobe-u.ac.jp) and CN (chikako@med.kobe-u.ac.jp)

Supplementary Figure Legends

Supplementary Figure S1. NIRS regression model dependent on the DNA concentration.

A) Y-fit for the DNA concentration using principal component regression (PCR) with pretreatment by mean centering, smoothing (21 points), OSC (one component), and active class validation. N = 32, number of applied latent variables = 3, r Cal = 0.9992, SEC = 0.2407, r Val = 0.9985, SECV = 0.3528.

B) Regression vector for the PLSR calibration model based on DNA concentration, showing characteristic water peaks in the 1400–1500 nm spectral interval.

Supplementary Figure S2. Regression vectors revealed characteristic water peaks using both the 1100–1850 and 1300–1850 nm intervals. Regression vectors of PLS-DA were used to identify nonirradiated and irradiated DNA solutions after spectral pretreatment by mean centering, smoothing (45 points), and OSC (one component) using the spectral intervals of 1100–1850 nm (A) and 1300–1600 nm (B).

Supplementary Figure S3. Results of the PCR calibration model for applied UVC irradiation doses of DNA solutions. Aqueous DNA solutions (20 μM) were irradiated with UVC at 0, 5, 10, 15, or 20 kJ/m². Strong correlations were found between the actual UVC irradiation dose and the level determined by the NIRS model in cross-validation.

A) Y-fit using PCR for UVC dose with pretreatment by mean centering, smoothing (21 points), OSC (one component), and leave-one-out cross-validation (r Val = 0.9550).

B) Characteristic water peaks were found at approximately 1400–1500 nm in the regression vector of PCR.

Supplementary Figure S4. Results of the PCR calibration model for the concentration of the produced cis-syn T<>Ts in DNA solutions.

Aqueous DNA solutions (20 μM) were irradiated with UVC at 0, 5, 10, 15, or 20 kJ/m^2. The concentrations of cis-syn T<>Ts produced in irradiated DNA solutions were determined by HPLC. Accurate NIRS calibration models were generated using laboratory reference values.

A) Y-fit using PCR with mean centering, smoothing (21 points), OSC (one component), and leave-one-out cross-validation for cis-syn T<>T concentrations (r Val 0.9399).

B) Characteristic water peaks were found at approximately 1400–1500 nm in regression vectors for PCR.

C) Graphs plotting the average predicted T<>T concentration using NIRS with PCR as a function of the UVC dose (R^2 = 0.956).

Supplementary Figure S5. Schema of the experiment.

Supplementary Fig. S1

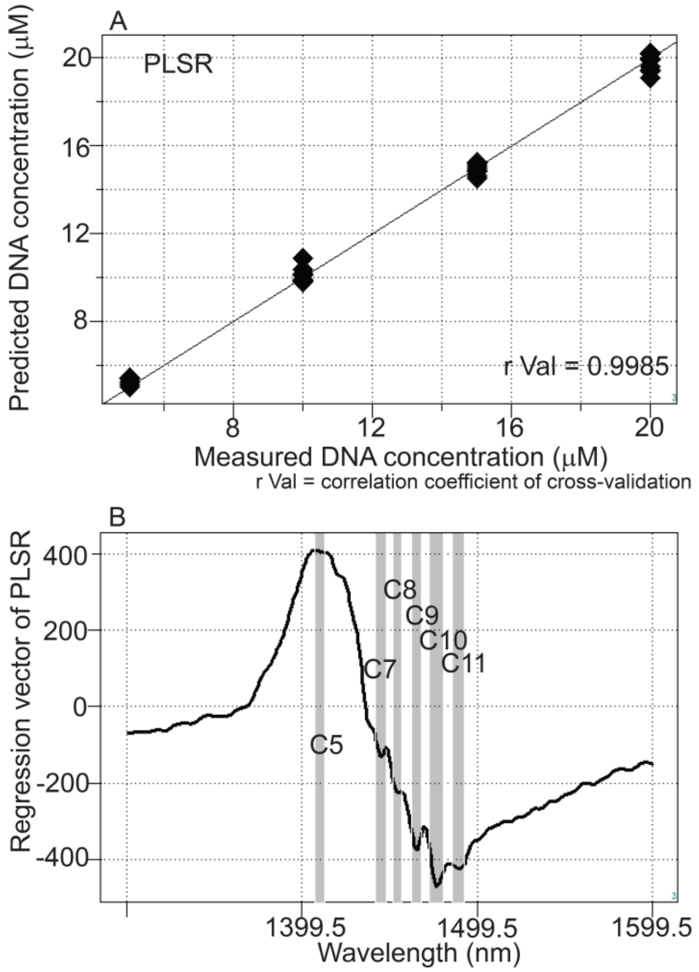

Supplementary Fig. S2

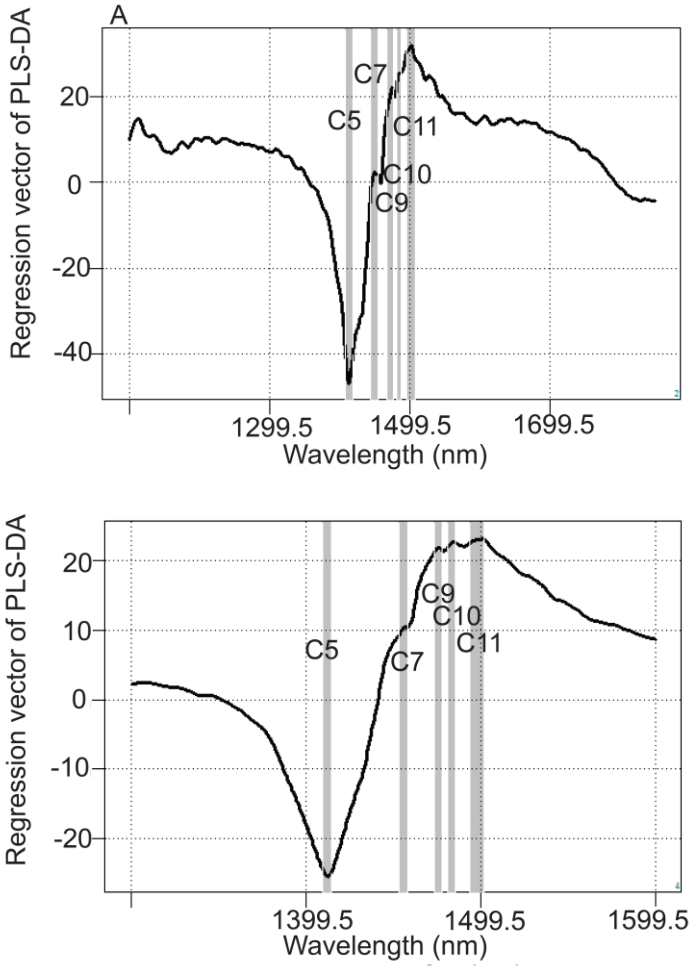

Supplementary Fig. S3

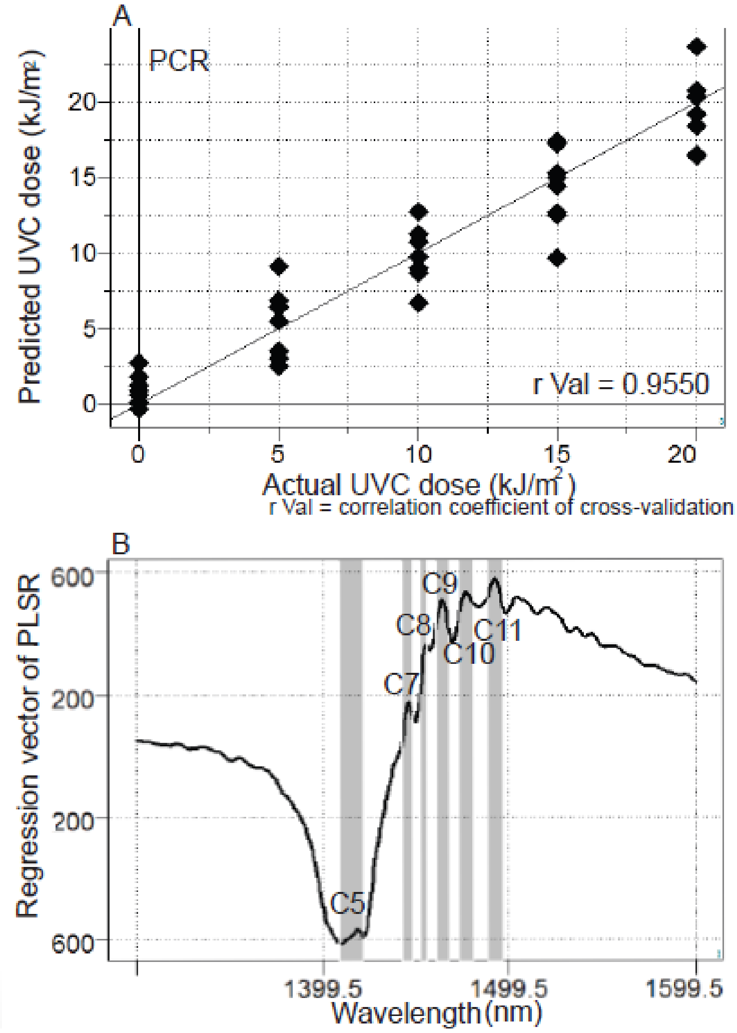

Supplementary Fig. S4

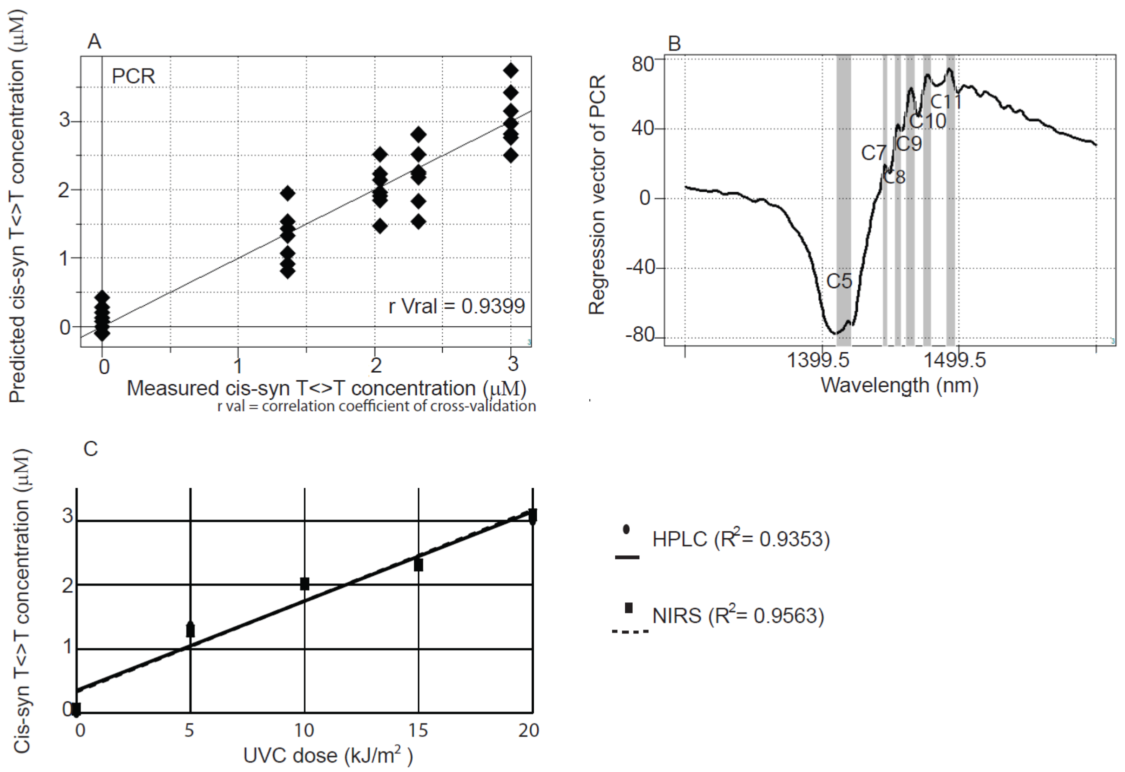

Supplementary Fig. S5

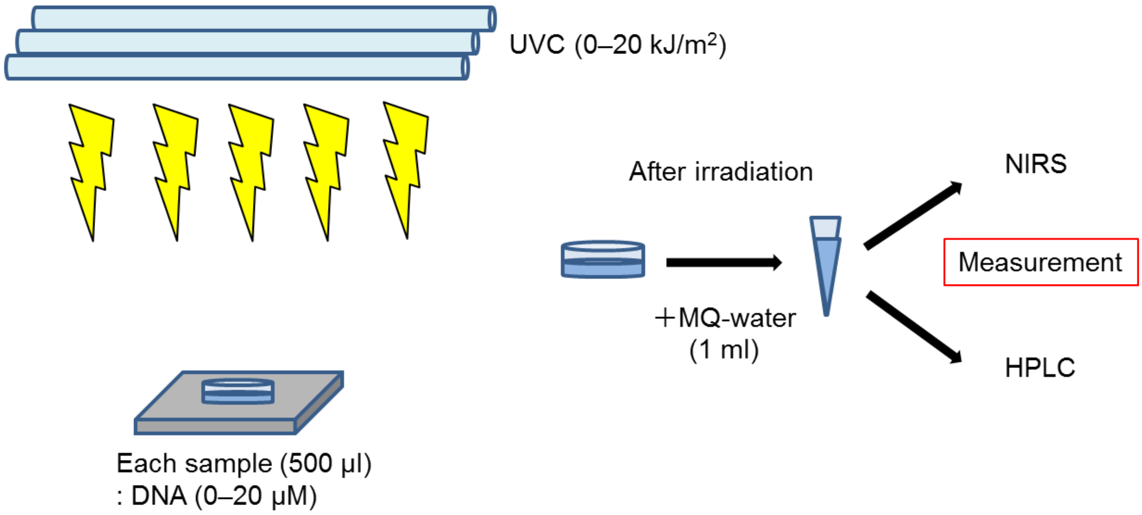

Supplementary Table S1. Calibration and cross-validation results of PLSR and PCR models for T<>T production rates at each DNA concentration.

	DNA concentration			
	**10 μM**		**20 μM**	
**N**	**39**		**39**	
	**PLS**	**PCR**	**PLS**	**PCR**
**Factor #**	2	2	3	2
**r Cal**	0.8906	0.8905	0.9565	0.9532
**SEC**	2.5766	2.5782	1.5890	1.6239
**r Val**	0.8685	0.8694	0.9472	0.9399
**SECV**	2.6989	2.7900	1.7482	1.7612

N = sample number, Factor # = number of latent variables, r Cal = correlation coefficient of calibration, SEC = standard error of calibration, r Val = correlation coefficient of cross-validation, SECV = standard error of cross-validation
